# Feline Calicivirus Infection Disrupts Assembly of Cytoplasmic Stress Granules and Induces G3BP1 Cleavage

**DOI:** 10.1128/JVI.00647-16

**Published:** 2016-06-24

**Authors:** Majid N. Humoud, Nicole Doyle, Elizabeth Royall, Margaret M. Willcocks, Frederic Sorgeloos, Frank van Kuppeveld, Lisa O. Roberts, Ian G. Goodfellow, Martijn A. Langereis, Nicolas Locker

**Affiliations:** a University of Surrey, Faculty of Health and Medical Sciences, School of Biosciences and Medicine, Guildford, United Kingdom; b Division of Virology, Department of Pathology, University of Cambridge, Addenbrooke's Hospital, Hills Road, Cambridge, United Kingdom; c Virology Division, Department of Infectious Diseases and Immunology, Faculty of Veterinary Medicine, Utrecht University, Utrecht, The Netherlands; Instituto de Biotecnologia/UNAM

## Abstract

In response to stress such as virus infection, cells can stall translation by storing mRNAs away in cellular compartments called stress granules (SGs). This defense mechanism favors cell survival by limiting the use of energy and nutrients until the stress is resolved. In some cases it may also block viral propagation as viruses are dependent on the host cell resources to produce viral proteins. Human norovirus is a member of the Caliciviridae family responsible for gastroenteritis outbreaks worldwide. Previous studies on caliciviruses have identified mechanisms by which they can usurp the host translational machinery, using the viral protein genome-linked VPg, or regulate host protein synthesis through the mitogen-activated protein kinase (MAPK) pathway. Here, we examined the effect of feline calicivirus (FCV) infection on SG accumulation. We show that FCV infection impairs the assembly of SGs despite an increased phosphorylation of eukaryotic initiation factor eIF2α, a hallmark of stress pathway activation. Furthermore, SGs did not accumulate in FCV-infected cells that were stressed with arsenite or hydrogen peroxide. FCV infection resulted in the cleavage of the SG-nucleating protein Ras-GTPase activating SH3 domain-binding protein (G3BP1), which is mediated by the viral 3C-like proteinase NS6^Pro^. Using mutational analysis, we identified the FCV-induced cleavage site within G3BP1, which differs from the poliovirus 3C proteinase cleavage site previously identified. Finally, we showed that NS6^Pro^-mediated G3BP1 cleavage impairs SG assembly. In contrast, murine norovirus (MNV) infection did not impact arsenite-induced SG assembly or G3BP1 integrity, suggesting that related caliciviruses have distinct effects on the stress response pathway.

**IMPORTANCE** Human noroviruses are a major cause of viral gastroenteritis, and it is important to understand how they interact with the infected host cell. Feline calicivirus (FCV) and murine norovirus (MNV) are used as models to understand norovirus biology. Recent studies have suggested that the assembly of stress granules is central in orchestrating stress and antiviral responses to restrict viral replication. Overall, our study provides the first insight on how caliciviruses impair stress granule assembly by targeting the nucleating factor G3BP1 via the viral proteinase NS6^Pro^. This work provides new insights into host-pathogen interactions that regulate stress pathways during FCV infection.

## INTRODUCTION

During infection by viruses, the accumulation of RNA replication intermediates or viral proteins imposes major stresses on the host cell. In response to these stresses, infected cells induce several defense mechanisms, which include the stress response pathways and the type I interferon (IFN) pathway. In order to promote cell survival and limit the use of energy and nutrients, the stressed host cell induces a global reduction in host protein synthesis (1). This translational arrest can be triggered by the phosphorylation of the eukaryotic initiation factor 2α (eIF2α) subunit, which prevents the recycling of the ternary complex ${\text{tRNA}}_{i}^{\text{Met}}$-GTP-eIF2 and the delivery of the initiator tRNA to the ribosome, thereby stalling the initiation of translation (1–4). While four kinases have been described that can induce eIF2α phosphorylation, protein kinase R (PKR) is specifically activated by double-stranded RNA (dsRNA) ligands present in the cytoplasm of virus-infected cells (1, 2, 5). Consequently, the inhibition of translation initiation leads to polysome disassembly, and the mRNAs contained within stalled ribosome complexes accumulate in cytoplasmic structures called stress granules (SGs) (4). The resulting inhibition of protein synthesis is problematic for viruses as they rely on the host cell protein synthesis machinery for production of their proteins. In turn, many viruses have evolved strategies to impair these cellular stress responses by disrupting SG formation during infection or exploiting SGs for their replication (6–10).

Although the exact composition of SGs remains elusive, their assembly is driven by aggregation-prone cellular RNA-binding proteins, such as T cell internal antigen 1 (TIA-1) and Ras-GTPase activating SH3 domain binding protein 1 (G3BP1), which bind to mRNAs, to one another, and subsequently to other cellular proteins, resulting in the formation of SGs (4). Furthermore, recent proteomic analysis of SG cores revealed that SG assembly is driven by an intricate network of protein-protein interactions modulated by ATPases (11). SGs contain mRNAs bound to translation factors, such as eIF4F and eIF3, and the 40S ribosomal subunit. They act as dynamic microdomains in which mRNAs are sorted between decay, storage, and polysome assembly, allowing cells to rapidly resume translation once stress is alleviated (4, 12, 13). Given that some viruses trigger the formation of SGs and translational shutoff through the phosphorylation of eIF2α (14, 15) and that RIG-I-like receptors and PKR have been shown to localize to SGs (16–18), it has been proposed that SGs could exert specific antiviral effects (19).

The Caliciviridae family contains small RNA viruses of both medical and veterinary importance. Human norovirus (HuNoV) is a leading cause of acute gastroenteritis worldwide, responsible for an estimated 18% of cases and 200,000 deaths per annum (20–23). The genogroup GII genotype 4 (GII.4) strains are responsible for the majority of outbreaks, including pandemics. While the symptoms are acute and self-resolving, HuNoV infection can result in inflammatory bowel disease or neonatal enterocolitis (24–26) and has been reported to cause persistent infections in young and elderly populations (27, 28). In animals, porcine sapovirus and bovine norovirus cause epidemic outbreaks of gastroenteritis in piglets and calves, respectively (29). Feline calicivirus (FCV), a member of the Vesivirus genus, causes upper respiratory tract infections and lethal systemic diseases in cats (30). Despite recent studies indicating that limited HuNoV replication can occur in immortalized B cells in the presence of enteric bacteria, a detailed understanding of human norovirus biology is limited owing to the lack of robust cell culture systems (31–33). However, the related caliciviruses murine norovirus (MNV) and FCV can be propagated in cell culture and remain the most robust and readily available models to understand the life cycle of caliciviruses (33, 34).

Members of the Caliciviridae family typically possess genomes ranging from 7.3 to 8.3 kb in length that have a viral genome-linked protein (VPg) covalently attached at the 5′ end. The VPg protein interacts with eIFs and acts a proteinaceous cap substitute (35, 36). While FCV VPg interacts with eIF4E to direct translation, in MNV it is the VPg interaction with eIF4G that is important for viral translation (35, 36). In addition, we have recently proposed that the control of eIF4E activity by the mitogen-activated protein kinase (MAPK) pathways contributes to calicivirus infection by regulating the translation of specific host mRNAs implicated in the antiviral response (37).

Despite these advances in our understanding of how caliciviruses manipulate the host translation machinery, the importance of stress response pathway activation and SGs in the calicivirus life cycle has not been addressed. Here, we examined the impact of FCV infection on SG accumulation. We observed that FCV infection impaired the assembly of SGs induced in an eIF2α-dependent and -independent manner. We show that this inhibition coincides with the cleavage of G3BP1 by the FCV protease NS6 at position 405. This study sheds new light on how caliciviruses manipulate the stress response pathway and possibly the innate antiviral responses.

## MATERIALS AND METHODS

### Cells and viruses.

Crandell-Rees feline kidney (CRFK) cells (European Collection of Cell Cultures [ECACC]) were grown in minimum essential medium (MEM) with Earle's salts and l-glutamine (Gibco), supplemented with 5% fetal bovine serum (FBS), 1% nonessential amino acids, and 1% (vol/vol) penicillin-streptomycin (5,000 units/ml penicillin G sodium and 5,000 μ/ml streptomycin sulfate in 0.85% saline; Life Technologies) in a 5% CO_2_ environment. Feline embryo fibroblast (FEA) cells (ECACC) were grown in MEM with Earle's salts and l-glutamine (Gibco), supplemented with 10% FBS, 1% nonessential amino acids, and 1% (vol/vol) penicillin-streptomycin (5,000 units/ml penicillin G sodium and 5,000 μg/ml streptomycin sulfate in 0.85% saline; Life Technologies) in a 5% CO_2_ environment. Murine macrophage cells J774.1 (ECACC) were maintained in Dulbecco's modified Eagle's medium (DMEM; Invitrogen) supplemented with 10% fetal calf serum (FCS; HyClone), 100 U of penicillin/ml, 100 μg of streptomycin/ml, 10 mM HEPES, and 2 mM l-glutamine in a 5% CO_2_ environment. The feline calicivirus (FCV) strain Urbana and murine norovirus 1 (MNV1) strain CW1 were described previously (35). Virus titers were estimated by determination of the 50% tissue culture infectious dose (TCID_50_) in units per milliliter. For a multiplicity of infection (MOI) equal to 1, cells were infected with 1 TCID_50_ unit per cell. Infection of CRFK or FEA cells and of J774 cells was carried out using multiplicities of infection (MOIs) of 1 and 10, respectively. The times postinfection refer to the time elapsed following medium replacement after a 1-h inoculation period. HEK293T cells were maintained in DMEM (Lonza) supplemented with 10% FCS and penicillin-streptomycin (100 U/ml and 100 μg/ml; ThermoFisher).

### G3BP1 KO engineering.

HeLa-R19-G3BP1^KO^ (with knockout [KO] of G3BP1) cells were generated using the CRISPR/Cas9 system as previously described (38). Briefly, genomic RNA (gRNA) encoding primer cassettes to target human G3BP1 (gRNA1, 5′-ACCGTAGTCCCCTGCTGGTCGGGC-3′ and 5′-AACGCCCGACCAGCAGGGGACTAC-3′; gRNA2, 5′-CCGTATTACACACTGCTGAACCG-3′ and 5′-AAACGGTTCAGCAGTGTGTAATA-3′) were cloned into the SapI restriction sites of the pCRISPR-hCas9-2xgRNA-Puro plasmid. HeLa-R19 cells were seeded in six-well clusters (100,000 cells/well) and transfected the next day with 2.5 μg of plasmid DNA using FuGENE6 (Promega) according to the manufacturer's instructions. The following day, successfully transfected cells were selected using puromycin, and single-cell clones were generated using endpoint dilutions. HeLa-R19-G3BP1^KO^ cells were grown in DMEM (Lonza) supplemented with 10% FCS and penicillin-streptomycin (100 U/ml penicillin G sodium and 100 μg/ml; ThermoFisher) in a 5% CO_2_ environment.

### Expression plasmids.

Construction of the pCMV-Flag-G3BP1 (where CMV is cytomegalovirus) and pCMV-Flag-G3BP2 plasmids is described elsewhere (39). Expression plasmids of mutant Flag-G3BP1 were developed using site-directed mutagenesis (SDM), and gene integrity was confirmed by sequence analysis. The pIRES-EGFP-MCS (where IRES is internal ribosome entry site, EGFP is enhanced green fluorescent protein, and MCS is multiple cloning site) was constructed by ligating a primer cassette (5′-GTACAAGCTCGAGATATATGCGGCCGCCTAAT-3′ and 5′-CTAGATTAGGCGGCCGCATATATCTCGAGCTT-3′; restriction sites are underlined) encoding XhoI-NotI restriction sites into the BsrGI-XbaI restriction sites of pIRES2-EGFP (Clontech). Coding sequences for FCV-NS6^Pro^, MNV-NS6^Pro^, and poliovirus (PV)-3C^Pro^ were amplified by PCR using primers possessing flanking XhoI and NotI restriction sites. Digested PCR products were ligated into the pIRES-EGFP-MCS plasmid, and gene integrity was confirmed by sequence analysis.

### Overexpression of viral proteinases.

HEK293T cells were seeded in six-well clusters (3.0 × 10^5^ cells/well) and the next day cotransfected with a pCMV-Flag-G3BP1 or pCMV-Flag-G3BP2 expression plasmid together with a pIRES-EGFP expression plasmid (ratio of 1:1; 2.5 μg/well) using FuGENE6 according to the manufacturer's instructions (Promega). At 16 h posttransfection, cells were collected, washed once with TEN-H buffer (40 mM Tris-HCl, pH 7.4, 150 mM NaCl, 10 mM EDTA) and lysed in TEN-L lysis buffer (40 mM Tris-HCl, pH 7.4, 150 mM NaCl, 10 mM EDTA, 1% NP-40, Complete Mini protease inhibitor cocktail [Roche]). Lysates were incubated for 15 min on ice, and cell debris was cleared using centrifugation (15 min at 15,000 × *g*). Protein concentration was determined using a bicinchoninic acid (BCA) assay (Pierce), and proteins were subjected to sodium dodecyl sulfate-polyacrylamide gel electrophoresis (SDS-PAGE).

### Induction of SGs.

To induce SG formation, cells were treated with 0.5 mM sodium arsenite (Sigma) for 30 min (CRFK and HeLa cells) or 45 min (J774 cells) or 50 μM for 1h (HeLa cells) or with 1 mM hydrogen peroxide (Sigma) for 1 h.

### Preparation and analysis of whole-cell extracts.

Cells were washed twice with cold phosphate-buffered saline (PBS) and then scraped into lysis buffer (50 mM HEPES, pH 7.4, 150 mM NaCl, 2 mM EDTA, 2 mM Na_3_VO_4_, 25 mM disodium β-glycerophosphate, Complete Mini protease inhibitor cocktail [1 tablet per 50 ml of buffer; Roche], 0.5% NP-40). Cell lysates were incubated on ice for 5 min before centrifugation at 14,000 × *g* for 5 min at 4°C in a microcentrifuge (Mikro 22R; Hettich Zentrifugen). For immunoblotting analysis during infection, standard protocols were used to separate cell lysates by SDS-PAGE, using 5 to 20 μg of extracts, and proteins were transferred to polyvinylidene difluoride membranes. These were probed with the primary antibodies G3BP1 (611126; BD Biosciences,), G3BP2 (A302-040A; Bethyl), MNV1 NS7 (40), FCV NS6/7 (35), poly(A)-binding protein (PAPB) (sc-28834; Santa Cruz), 4E-BP1 Ser65 (9451; Cell Signaling), 4E-BP1 Thr37/46 (9459; Cell Signaling,), 4E-BP1 (9452; Cell Signaling,), glyceraldehyde-3-phosphate dehydrogenase (GAPDH) (AM4300; Ambion), eIF2α (9722; Cell Signaling), and eIF2α Ser52 (44-728G; Invitrogen), followed by incubation with the appropriate peroxidase-labeled secondary antibody (Dako) and chemiluminescence development using SuperSignal West Pico chemiluminescence substrate (Pierce). The signals were detected on radiographic film (Fuji; New England BioLabs). For immunoblotting analysis during overexpression experiments, membranes were successively incubated for 1 h with the primary antibody G3BP1, G3BP2, Flag-M2 (F3165; Sigma-Aldrich), EGFP (OSE00003G; Invitrogen), or tubulin (T6074; Sigma-Aldrich) and then for 30 min with goat anti-mouse labeled with IRDye 680 (Li-Cor) or goat anti-rabbit labeled with IRDye 800 (Li-Cor). The signals were detected by scanning the membranes using an Odyssey Imager (Li-Cor).

### Immunofluorescence microscopy.

J774 cells grown on poly-l-lysine-treated coverslips were infected with MNV1 at an MOI of 10 and fixed for 15 min with 4% paraformaldehyde in PBS at the time points indicated on the figures. For arsenite stress, cells were treated with 0.5 mM sodium arsenite for 45 min before fixation and immunostaining. Cells were then permeabilized for 5 min with PBS–0.2% Triton X-100, and unspecific antigens were blocked for 1 h using 2% normal goat serum (S2007; Sigma) in PBS–0.1% Tween 20 (PBST). Cells were then incubated for 1 h with primary antibodies in PBST at a dilutions 1:2,000 (anti-NS3 MNV; noncommercial rabbit polyclonal) and 1:500 (anti-G3BP1, mouse monoclonal [611126; BD Biosciences]). After extensive washes with PBST, species-matched Alexa Fluor-conjugated secondary antibodies (A-11037 and A-11029; ThermoFisher Scientific) were added at a dilution of 1:500 in PBST for one additional hour. Coverslips were extensively washed and mounted on slides with Mowiol supplemented with 4′,6′-diamidino-2-phenylindole (DAPI) and 1,4-diazabicyclo[2.2.2]octane (DABCO). Confocal micrographs were acquired on a Leica TCS SP5 confocal microscope fitted with a 63× (1.3 numerical aperture [NA]) oil immersion objective using 488-nm and 594-nm laser excitation lines under sequential channel scanning to prevent fluorophore bleed-through artifacts due to spectral overlap.

CRFK cells grown on coverslips were infected with FCV Urbana at an MOI of 1 and fixed for 1 h with 4% paraformaldehyde in PBS at the time points indicated on the figures. Cells were then permeabilized for 5 min with PBS–0.1% Triton X-100, and unspecific antigens were blocked for 30 min using 1% bovine serum albumin (BSA) in PBS (PBS-BSA). Cells were then incubated for 1 h with primary antibodies in PBS-BSA at dilutions of 1:150 (anti-eIF4G, rabbit polyclonal [sc-11373; Santa-Cruz]), 1:400 (anti-G3BP1, mouse monoclonal [611126; BD biosciences]), 1:600 (anti-NS6/7, rabbit polyclonal [35]), and 1:10,000 (anti-VP1, mouse polyclonal [35]). After extensive washes with PBS, species-matched Alexa Fluor-conjugated secondary antibodies (A-21424 and A-11034; ThermoFisher Scientific) were added at a dilution of 1:400 in PBS-BSA for one additional hour. Coverslips were extensively washed and then incubated in To-Pro-3 (T3605; ThermoFisher Scientific) for 10 min before being mounted on slides with Vectashield (H1000; Vector Laboratories). Confocal micrographs were acquired on a Zeiss LSM510 VIS Meta confocal microscope fitted with a 40× (1.3 NA) oil immersion objective using 488-nm, 594-nm, and 633-nm laser excitation lines under sequential channel scanning to prevent fluorophore bleed-through artifacts due to spectral overlap.

HeLa-R19-G3BP1^KO^ cells were grown on coverslips in 24-well clusters and the next day cotransfected with a pCMV-Flag-G3BP1 or pCMV-Flag-G3BP1-E^405^A expression plasmid together with a pIRES-EGFP or pIRES-EGFP-FCV-NS6 expression plasmid (ratio of 4:1; 0.5 μg/well) using FuGENE6 according to the manufacturer's instructions (Promega). The following day, cells were treated with 50 μM arsenite for 1 h and fixed for 30 min with 4% paraformaldehyde in PBS. Cells were then permeabilized for 5 min with PBS–0.2% Triton X-100, and unspecific antigens were blocked for 1 h using 2% BSA in PBS (PBS-BSA). Cells were then incubated for 1 h with primary antibodies in PBS-BSA at dilutions of 1:500 (anti-eIF3η, goat polyclonal [sc-16377; Santa-Cruz]) and 1:1,000 (anti-G3BP1, mouse monoclonal [611126; BD Biosciences]). After extensive washes with PBS, species-matched Alexa Fluor-conjugated secondary antibodies (A-11058 and A-31571; ThermoFisher Scientific) were added at a dilution of 1:100 with DAPI (1 μg/ml) in PBS-BSA for 30 min. Coverslips were extensively washed and mounted on glass slides in FluorSafe (Calbiochem). Cells were examined by confocal microscopy (Leica SPE-II).

For the quantification of SGs, a minimum of 500 cells for three independent replicates were analyzed. Cells were considered SG positive if two or more SG marker foci were present in the cytoplasm. Statistical analyses were then performed using GraphPad software. All error bars represent standard deviations calculated from a minimum of three independent biological replicates. Analysis of variance (ANOVA) was applied to the data sets to calculate *P* values.

## RESULTS

### eIF2α is phosphorylated in FCV-infected cells.

**FIG 1:**
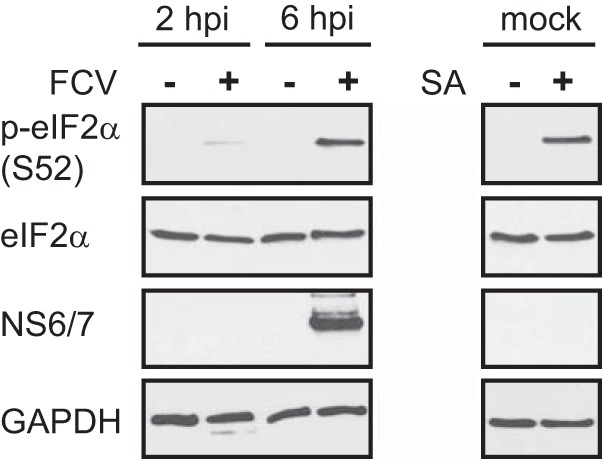
eIF2α phosphorylation during FCV infection. CRFK cells were mock infected or infected with FCV Urbana at an MOI of 1 for 2 or 6 h as indicated. As a control, CRFK cells were treated with 0.5 mM sodium arsenite (SA; +) for 30 min or mock treated (−). Following treatments, cell extracts were prepared and analyzed by SDS-PAGE and immunoblotting using the antibodies indicated on the left side of the panel. p-eIF2α, phospho-eIF2α.

Rapidly after infection, the accumulation of viral products, such as RNA replication intermediates or viral proteins, acts as the trigger for the cellular stress response pathways (1, 2). The recognition of viral RNA by PKR promotes the phosphorylation of the translation factor eIF2α and elicits potent innate immune responses (3). To examine the effect of FCV infection on the host cell, we monitored the levels of phosphorylated eIF2α. To this end, the lysates from mock- and FCV-infected CRFK cells, at 2 or 6 h postinfection (hpi), were fractionated by SDS-PAGE and analyzed by immunoblotting. As shown on Fig. 1, the levels of total eIF2α were not affected during infection. However, FCV infection resulted in eIF2α phosphorylation at 2 and 6 hpi, a similar response to that seen following sodium arsenite treatment, a potent inducer of eIF2α phosphorylation via the kinase HRI (41).

### FCV infection does not cause accumulation of SGs.

**FIG 2:**
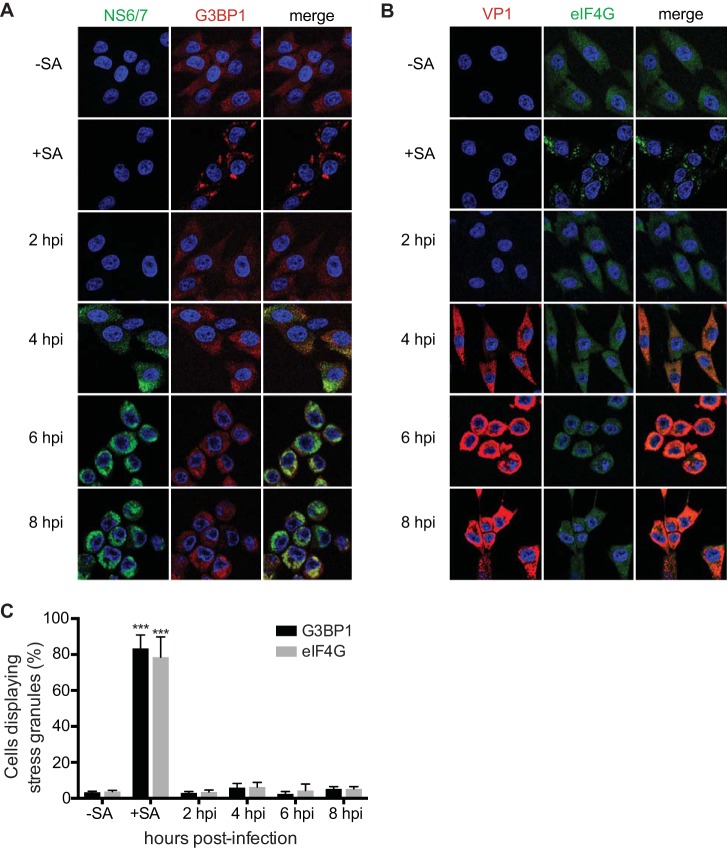
SG accumulation during FCV infection. (A) CRFK cells were mock infected or infected with FCV Urbana at an MOI of 1. Infected cells, with (+) or without (−) sodium arsenite (SA) treatment, were fixed at the times indicated postinfection and stained with either mouse monoclonal antibodies specific for G3BP1 and rabbit polyclonal antiserum specific for FCV NS6/7 (A) or mouse polyclonal antibodies specific for VP1 and rabbit polyclonal antiserum specific for eIF4G (B). This was followed by staining with Alexa Fluor 488-conjugated donkey anti-rabbit IgG and Alexa Fluor 555-conjugated donkey anti-mouse IgG secondary antibodies. Nuclei were stained with To-Pro-3. Stained cells were examined by fluorescence microscopy, and representative images are shown. (C) The percentage of cells containing SGs out of the total number of cells was calculated, and the means of and standard deviations of three experimental replicates are shown. Results were analyzed by one-way ANOVA with Bonferroni corrections: ***, *P* < 0.001 (GraphPad Prism, version 6).

eIF2α phosphorylation prevents the recycling of the ternary complex ${\text{tRNA}}_{i}^{\text{Met}}$-GTP-eIF2 and the delivery of the initiator tRNA to the ribosome, thereby stalling the initiation of translation and resulting in a shutdown of protein synthesis. Following this translational arrest, mRNAs contained within stalled ribosome complexes accumulate in cytoplasmic structures called SGs (4). Therefore, we investigated whether FCV infection also resulted in the formation of SGs. CRFK cells were infected for 8 h and then stained with antisera specific for NS6/7, a viral protein, and G3BP1, a marker of SGs, at various times postinfection. Cells were then examined by immunofluorescence microscopy to determine whether FCV-infected cells contained SGs. We observed that, despite eIF2α phosphorylation, FCV infection failed to trigger SG accumulation throughout the replication cycle (Fig. 2A). The quantification of the number of cells displaying G3BP1 foci did not increase significantly during infection (Fig. 2C). To confirm these results, we also examined the accumulation of another SG marker, eIF4G, during FCV infection. Again, FCV infection did not result in an increase of eIF4G cytoplasmic foci (Fig. 2B and C). To investigate whether CRFK cells are able to form SGs upon stress pathway activation, CRFK cells were treated for 30 min with 0.5 mM arsenite. This treatment resulted in the relocalization of G3BP1 and eIF4G to discrete cytoplasmic foci (Fig. 2A and B). Thus, these data suggest that FCV infection does not induce the accumulation of SGs, despite the induction of eIF2α phosphorylation.

### FCV infection blocks the formation of SGs in response to arsenite treatment.

**FIG 3:**
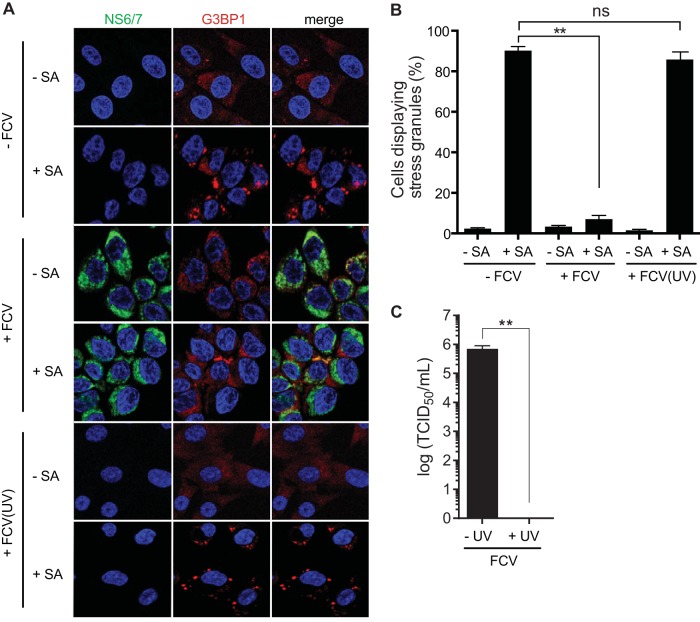
SG accumulation following arsenite treatment in FCV-infected cells. (A) CRFK cells were mock infected or infected with FCV Urbana at an MOI of 1 for 6 h. Following infection, cells were untreated (−) or treated (+) with 0.5 mM sodium arsenite (SA) for 30 min. The cells were fixed and stained with mouse monoclonal antibodies specific for G3BP1 and rabbit polyclonal antiserum specific for FCV NS6/7. This was followed by staining with Alexa Fluor 488-conjugated donkey anti-rabbit IgG and Alexa Fluor 555-conjugated donkey anti-mouse IgG secondary antibodies. Nuclei were stained with To-Pro-3. Stained cells were examined by fluorescence microscopy, and representative images are shown. (B) The percentage of cells containing SGs out of the total number of cells was calculated, and the means and standard deviations of three experimental replicates are shown. Results were analyzed by one-way ANOVA with Bonferroni corrections: **, *P* < 0.01; ns not significant (GraphPad Prism, version 6). (C) CRFK cells were infected with FCV or UV-inactivated FCV at an MOI of 1. The cells were incubated for 12 h, and the viral titer was estimated by a TCID_50_ assay. Three separate experiments were analyzed by standard *t* test (**, *P* < 0.01; GraphPad Prism, version 6). −FCV, mock infection; +FCV(UV), infection with UV-inactivated FCV.

The absence of SGs in FCV-infected CRFK cells, despite eIF2α phosphorylation, suggested that FCV infection could impair the assembly of SGs, potentially disconnecting the cellular signals that mediate the accumulation of SGs. To examine whether FCV infection could block SG formation in response to exogenous stress triggers, we monitored arsenite-induced SG formation in mock- and FCV-infected cells. CRFK cells were mock or FCV infected for 6 h, treated with arsenite for 30 min, and then fixed and stained for immunofluorescence microscopy. In mock-infected cells, arsenite-induced SGs were detected in 90% of cells (Fig. 3A and B). In contrast, no significant induction of SG assembly could be detected in FCV-infected cells (Fig. 3A and B). To determine whether this absence of SGs was due to viral replication, the experiment was repeated with UV-inactivated virus, which is replication incompetent (Fig. 3C). FCV inactivation restored the ability of infected cells to assemble SGs in response to arsenite treatment to levels similar to those in mock-infected cells (Fig. 3A and B). Thus, FCV replication impairs the formation of SGs in response to stress pathway activation.

### FCV infection impairs the assembly of hydrogen peroxide-induced SGs.

**FIG 4:**
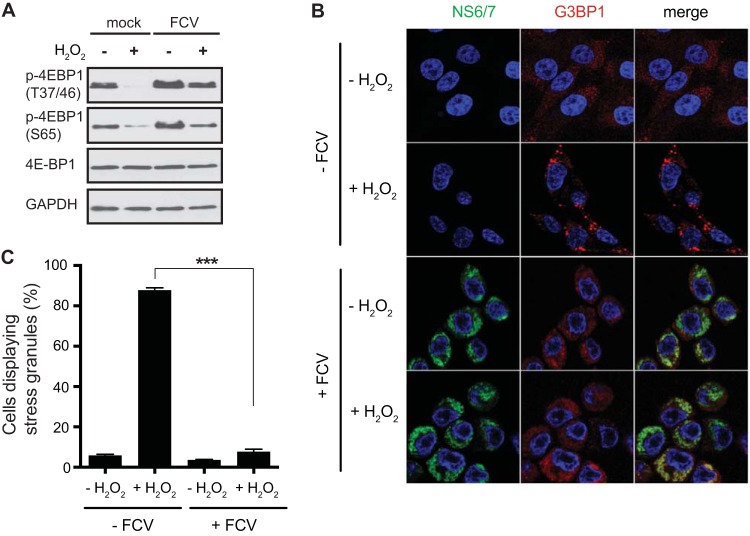
SG accumulation following hydrogen peroxide treatment in FCV-infected cells. (A) CRFK cells were mock infected or infected with FCV Urbana at an MOI of 1 for 6 h and treated with 1 mM hydrogen peroxide (H_2_O_2_; +) for 1 h or mock treated (−). Following treatments, cell extracts were prepared and analyzed by SDS-PAGE and immunoblotting using the antibodies indicated on the left side of the panels. (B) CRFK cells were mock infected (−FCV) or infected with FCV Urbana (+FCV) at an MOI of 1 for 6 h. Following infection, cells were treated with 1 mM H_2_O_2_ for 1 h. The cells were fixed and stained with mouse monoclonal antibodies specific for G3BP1 and rabbit polyclonal antiserum specific for FCV NS6/7. This was followed by staining with Alexa Fluor 488-conjugated donkey anti-rabbit IgG and Alexa Fluor 555-conjugated donkey anti-mouse IgG secondary antibodies. Nuclei were stained with To-Pro-3. Stained cells were examined by fluorescence microscopy, and representative images are shown. (C) The percentage of cells containing SGs out of the total number of cells was calculated, and the means and standard deviations of three experimental replicates are shown. Results were analyzed by one-way ANOVA with Bonferroni corrections: ***, *P* < 0.001 (GraphPad Prism, version 6).

The absence of SGs in FCV-infected CRFK cells following arsenite stress, despite the occurrence of eIF2α phosphorylation, suggested that FCV infection impairs SG formation. Next, we tested whether FCV infection would also interfere with the accumulation of SGs mediated by eIF2α-independent translational stalling. Previous studies have suggested that hydrogen peroxide stress results in 4EBP1 dephosphorylation, leading to eIF4E sequestration. This, in turn, induces the disassembly of the eIF4F complex, translational stalling, and SG accumulation (42). First, to confirm that hydrogen peroxide induced translation inhibition in our experimental system, CRFK cells were treated with hydrogen peroxide, and 4EBP1 phosphorylation was assessed by immunoblotting using antibodies specific for phosphorylated 4EBP1. Hydrogen peroxide treatment resulted in dephosphorylation of 4EBP1 at T36/47 and S65 (Fig. 4A). Given that S65 is one of the two main phosphorylation sites (along with T70) that controls the ability of 4EBP1 to sequester eIF4E, while the phosphorylation at T36/47 is not directly linked to eIF4E binding (43), we concluded that hydrogen peroxide stress of CRFK cells induces translational impairment, as expected. Next, mock- or FCV-infected CRFK cells were exposed at 6 hpi to hydrogen peroxide, and the accumulation of SGs was evaluated by immunofluorescence microscopy using G3BP1 as an SG marker. While 87.7% of mock-infected cells assembled SGs in response to hydrogen peroxide, FCV-infected cells were unable to assemble SGs (Fig. 4B and C). Together, these data suggest that FCV infection impairs the assembly of SGs that are triggered via eIF2α-dependent and independent pathways.

### FCV infection results in the cleavage of the SG-nucleating protein G3BP1.

**FIG 5:**
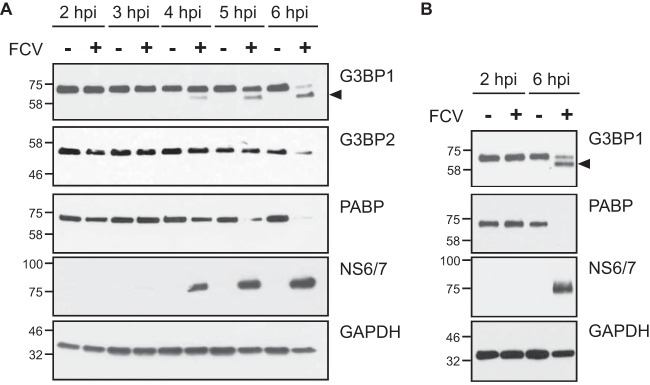
G3BP1 cleavage during FCV infection. CRFK (A) and FEA (B) cells were mock infected (−) or infected (+) with FCV Urbana at an MOI of 1 for 6 h. Cell extracts were prepared and analyzed by SDS-PAGE and immunoblotting using the antibodies indicated to the right of the panels. The arrows denote the positions of cleavage products, and the molecular mass standards (kilodaltons) are shown on the left.

The absence of SGs following translational arrest mediated by both eIF2α-dependent and -independent pathways suggested that FCV infection might impair the ability of cells to assemble SGs. Previous studies have demonstrated that SG inhibition could be driven by the sequestration of SG-nucleating proteins or the cleavage of the SG-nucleating component G3BP1 (8). For example, G3BP1 cleavage is mediated by the proteinase 3C^Pro^ of poliovirus (PV), a virus that belongs to the Picornaviridae family (44). Caliciviruses share a similar genome organization with picornaviruses and express a proteinase related to the 3C^Pro^, namely, 3C-like protease (3CL^Pro^) or NS6^Pro^ (45). In addition to cleaving viral proteins, NS6^Pro^ can target cellular proteins such as PABP to regulate translation during infection (46). Therefore, we investigated whether FCV infection could result in G3BP1 cleavage, explaining the absence of SG accumulation upon arsenite or hydrogen peroxide treatment. Lysates from mock- and FCV-infected CRFK cells were fractionated by SDS-PAGE and analyzed by immunoblotting. As shown in Fig. 5, FCV infection resulted in the degradation of PABP from 4 hpi while levels of GAPDH remained constant (Fig. 5A). In addition, from 4 hpi onwards, a smaller cleavage product from G3BP1 could be detected. At 6 hpi, most of the full-length G3BP1 was cleaved into the short form (Fig. 5A). We also analyzed the impact of FCV infection on G3BP2, a G3BP1 homolog protein recruited to SGs (47). Our results suggest that G3BP2 is also impacted by FCV infection (Fig. 5A) although this is only evident at 6 hpi. To confirm these results, we infected FEA cells with FCV. Similarly to the results observed with CRFK cells, infection of FEA cells with FCV resulted in the cleavage of G3BP1 into a shorter form (Fig. 5B). Of note, we could not detect the presence of G3BP2 in FEA cells (data not shown).

### The FCV, but not MNV, NS6^Pro^ cleaves G3BP1 and G3BP2.

**FIG 6:**
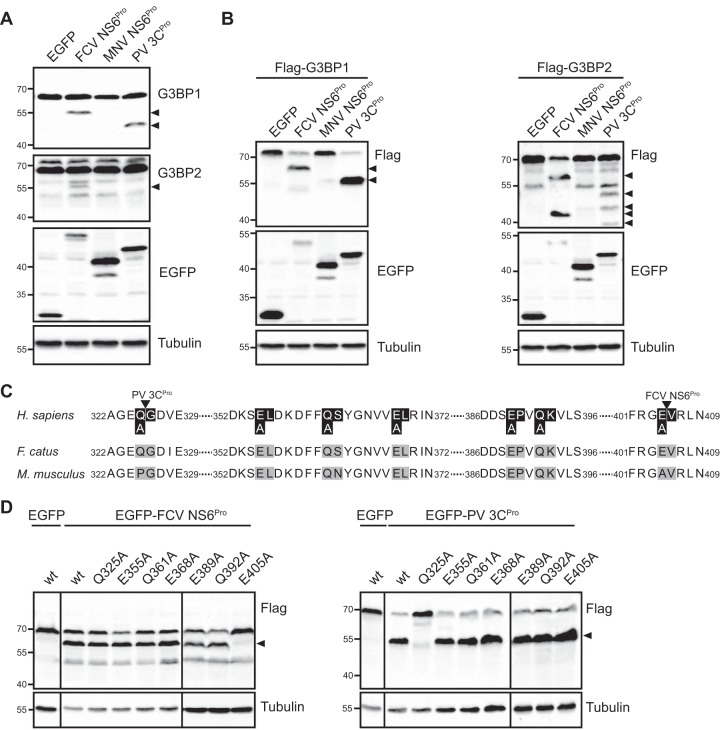
G3BP1 and G3BP2 cleavage by FCV NS6^Pro^. (A) EGFP, EGFP-FCV NS6^Pro^, EGFP-MNV NS6 ^Pro^, or EGFP-PV 3C ^Pro^ was expressed in 293T cells. Cell extracts were prepared and analyzed by SDS-PAGE and immunoblotting using the antibodies indicated to the right of the panels. The arrows denote the positions of cleavage products, and the molecular mass standards (kilodaltons) are shown on the left. (B) EGFP, EGFP-FCV NS6 ^Pro^, EGFP-MNV NS6 ^Pro^, or EGFP-PV 3C ^Pro^ was coexpressed with Flag-G3BP1 or Flag-G3BP2 in 293T cells, as indicated. Cell extracts were prepared and analyzed by SDS-PAGE and immunoblotting using the antibodies indicated to the right of the panels. The arrows denote the positions of cleavage products, and the molecular mass standards (kilodaltons) are shown on the left. (C) Comparison of the C-terminal sequences of human, feline, and murine G3BP1 proteins. The positions of the viral proteinase cleavage sites, identified in this study and previously, are indicated above the sequences boxed in black. The positions where G3BP1 alanine mutants have been introduced are highlighted in black and gray. (D) EGFP, EGFP-FCV NS6 ^Pro^, or EGFP-PV 3C^Pro^ was coexpressed with wild-type (wt) or mutant Flag-G3BP1 in 293T cells, as indicated. Cell extracts were prepared and analyzed by SDS-PAGE and immunoblotting using the antibodies indicated to the right of the panels. The arrows denote the positions of cleavage products, and the molecular mass standards (kilodaltons) are shown on the left. H. sapiens, *Homo* sapiens; F. catus, Felis catus; M. musculus, Mus musculus.

To further establish whether the FCV NS6^Pro^ is directly responsible for the cleavage of G3BP1 observed, we expressed the FCV NS6^Pro^ and PV 3C^Pro^ as EGFP fusion proteins in HEK293T cells. While the expression of EGFP alone had no impact on G3BP1, the expression of the EGFP-PV 3C^Pro^ resulted in the cleavage of endogenous G3BP1 (Fig. 6A). The expression of EGFP-FCV NS6^Pro^ also resulted in the cleavage of G3BP1; however, the cleavage product detected differed from the one generated by EGFP-PV 3C^Pro^ and more closely resembled the products observed in virus-infected cells (Fig. 6A). We also analyzed the impact of viral proteases on G3BP2, a G3BP1 homolog protein recruited to SGs (47). While EGFP-PV 3C^Pro^ had no impact on G3BP2 integrity, G3BP2 cleavage was detected in cells expressing EGFP-FCV NS6^Pro^. To confirm these findings, we analyzed the impact of EGFP-PV 3C^Pro^ and EGFP-FCV NS6^Pro^ on overexpressed Flag-G3BP1 and Flag-G3BP2 in HEK293T cells. Again, we observed that both EGFP-PV 3C^Pro^ and EGFP-FCV NS6^Pro^ were able to cleave the Flag-G3BP1 fusion protein and that the cleavage products were of different sizes (Fig. 6B). Moreover, Flag-G3BP2 was efficiently cleaved by EGFP-FCV NS6^Pro^ while EGFP-PV 3C^Pro^ expression resulted in only weak cleavage at several distinct sites (Fig. 6B). These results suggest that the impairment of SG assembly during FCV infection may be due to the NS6^Pro^-mediated cleavage of both G3BP1 and G3BP2. Next, we analyzed whether the expression of NS6^Pro^ from a related calicivirus would also impact G3BP1 and G3BP2 integrity. However, the expression of EGFP-MNV NS6^Pro^ did not result in cleavage of G3BP1 or G3BP2 (Fig. 6A and B). This suggests that this new pattern of G3BP1 and G3BP2 cleavages is restricted to FCV infection rather than being conserved in caliciviruses.

### FCV NS6^Pro^ cleaves G3BP1 at position E405.

Our results suggest that FCV NS6^Pro^ cleaves G3BP1 at a position that differs from the previously identified PV 3C^Pro^ cleavage at Q325 (Fig. 6C). To confirm this, we overexpressed wild-type (wt) or Flag-G3BP1 with the Q325A substitution and analyzed the impact on EGFP-FCV NS6^Pro^ cleavage efficacy. As previously shown, the Q325A substitution inhibited the cleavage mediated by EGFP-PV 3C^Pro^ (7); however, this did not prevent cleavage mediated by EGFP-FCV NS6^Pro^ (Fig. 6D), confirming that FCV NS6^Pro^ and PV 3C^Pro^ cleave G3BP1 at distinct positions. Previous studies have shown that FCV NS6^Pro^ could induce proteolysis at the following P1-P1′ junctions: E/A, D/A, Q/A, E/V, E/R, E/L, E/G, and E/H (48). To further determine the cleavage site, we replaced all Q and E residues with A residues in the G3BP1 C-terminal region, yielding a cleavage product of around 8 to 12 kDa (Fig. 6C). The impact of these substitutions was then evaluated by monitoring wild-type or mutant Flag-G3BP1 integrity during the coexpression with EGFP-FCV NS6^Pro^. While the E355A, Q361A, E368, E389A, and Q392A substitutions did not prevent G3BP1 cleavage, the E405A substitution rendered Flag-G3BP1 resistant to EGFP-FCV NS6^Pro^ cleavage (Fig. 6D). These results suggest that FCV NS6^Pro^ mediates the cleavage of G3BP1 at E405/V406.

### FCV NS6^Pro^-mediated G3BP1 cleavage impairs SG assembly.

**FIG 7:**
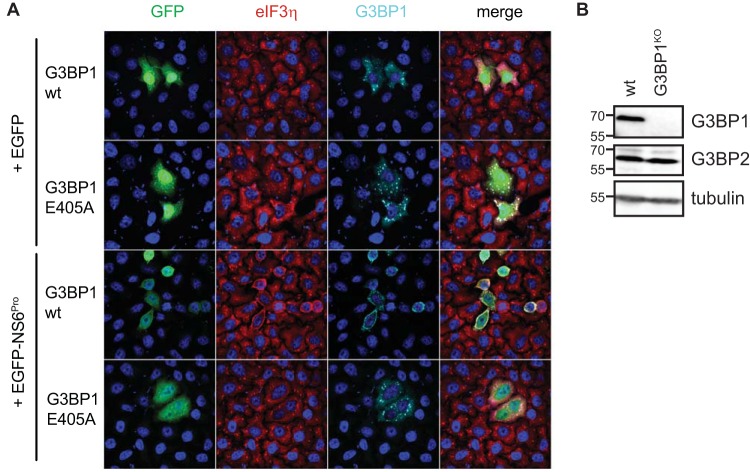
FCV NS6^Pro^-mediated G3BP1 cleavage impairs SG assembly. (A) HeLa-R19-G3BP1^KO^ cells were cotransfected with a Flag-G3BP1 or Flag-G3BP1-E405A expression plasmid together with an EGFP or EGFP-FCV-NS6^Pro^ expression plasmid and treated with 50 μM sodium arsenite for 1 h. Cells were then fixed and stained with goat polyclonal antibodies specific for eIF3η and mouse monoclonal specific for G3BP1. This was followed by staining with species-matched Alexa Fluor-conjugated secondary antibodies. Nuclei were stained with DAPI. Stained cells were examined by fluorescence microscopy, and representative images are shown. (B) Cell extracts were prepared from HeLa (wt) and HeLa-R19-G3BP1^KO^ (G3BP1^KO^) cells and analyzed by SDS-PAGE and immunoblotting using the antibodies indicated on the right side of the panels. The molecular mass standards (kilodaltons) are shown on the left.

To confirm that G3BP1 cleavage at E405 by FCV NS6^Pro^ would indeed impair SG formation, we analyzed the impact of the E405A mutation on FCV NS6^Pro^-mediated inhibition of SG formation. First, we confirmed that both Flag-G3BP1 wt and E405A localize to SGs in HeLa-R19-G3BP1^KO^ cells generated by the CRISPR/Cas9 system (Fig. 7A and B). Coexpression of Flag-G3BP1 wt or Flag-G3BP1 E405A, together with an EGFP control, in the presence of sodium arsenite resulted in the accumulation of both Flag-G3BP1 wt and Flag-G3BP1 E405A in cytoplasmic foci that are also positive for eIF3. The colocalization of the tagged G3BP1 with eIF3 strongly suggests that the recombinant Flag-G3BP1 proteins accumulate in SGs and that the E405A mutation does not affect SG formation (Fig. 7A). As expected, the coexpression of Flag-G3BP1 wt with EGFP-FCV NS6^Pro^ in the presence of sodium arsenite resulted in diffused cytoplasmic localization of Flag-G3BP1 wt, reflecting an impaired ability to assemble SGs when G3BP1 is cleaved by FCV NS6^Pro^. In stark contrast, the coexpression of Flag-G3BP1 E405A with EGFP-FCV NS6^Pro^ in the presence of sodium arsenite restored the ability of cells to assemble SGs, as noted by the accumulation of G3BP1 foci (Fig. 7A). Thus, G3BP1 cleavage at E405 by the FCV NS6^Pro^ is sufficient to impair SGs accumulation.

### MNV infection does not impair SG assembly following a stress trigger.

**FIG 8:**
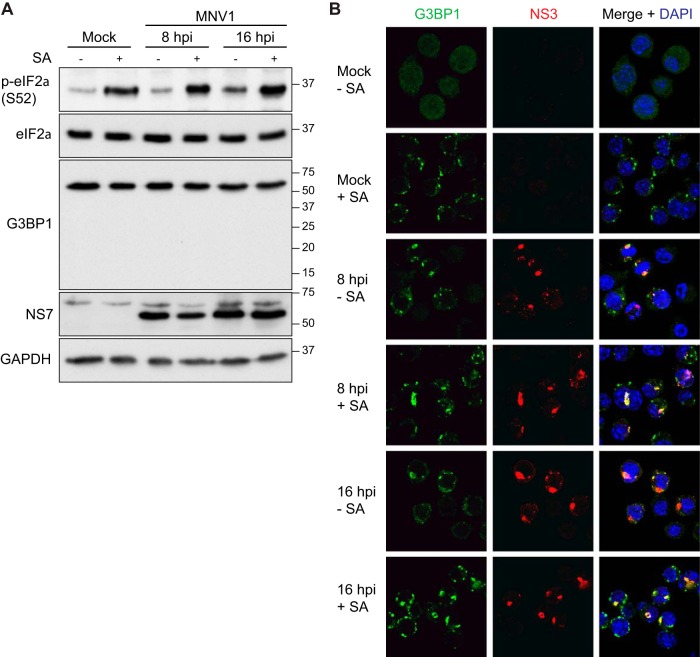
SG accumulation during MNV infection. (A) J774 cells were mock infected or infected with MNV at an MOI of 10 and untreated (−) or treated (+) with 0.5 mM sodium arsenite (SA) for 45 min. Cell extracts were prepared and analyzed by SDS-PAGE and immunoblotting using the antibodies indicated on the left side of the panels. The molecular mass standards (kilodaltons) are shown on the right. (B) J774 cells were mock infected or infected with MNV1 at an MOI of 10. Following infection cells were untreated or treated with 0.5 mM SA for 45 min. Cells were fixed at the times indicated postinfection and stained with rabbit polyclonal antibodies specific for NS3 and mouse monoclonal antibodies specific for G3BP1. This was followed by staining with species-matched Alexa Fluor-conjugated secondary antibodies.

In the absence of G3BP1 cleavage by MNV NS6^Pro^ (Fig. 6A), we hypothesized that MNV-infected cells would be able to assemble SGs in response to arsenite treatment. To test this, murine J774 cells were infected with MNV for 8 or 16 h, followed by immunoblotting analysis. At both 8 and 16 hpi, no G3BP1 cleavage product could be detected (Fig. 8A). Furthermore, MNV infection of J774 cells resulted in eIF2α phosphorylation, which increased during the late stages of the viral life cycle, i.e., at 8 to 16 hpi (Fig. 8A). The formation of SGs at 8 and 16 hpi was then analyzed by immunofluorescence microscopy using G3BP1 as an SG marker and NS3 as a marker for viral protein accumulation. J774 cells can assemble SGs in response to sodium arsenite treatment, as evidenced by the accumulation of G3BP1 foci (Fig. 8B). In contrast with FCV infection, MNV infection resulted in the assembly of SGs (Fig. 8B). Interestingly, some of these G3BP1 foci seem to colocalize with NS3. Given that MNV NS3 accumulates in replication complexes during infection, further studies will aim at dissecting a putative interaction between SGs and replication complexes (49). Importantly, MNV infection did not impair the accumulation of SGs in MNV-infected cells stimulated with sodium arsenite at either 8 or 16 hpi. Therefore, unlike FCV, MNV infection does not impair the ability of cells to form SGs, at least in the culture system examined here, and we propose that this is due to the absence of G3BP1 cleavage.

## DISCUSSION

Viral infections cause major stress for the infected host cell that in many cases can culminate in translational arrest. The stress-induced translational repression is a consequence of reduced translation initiation that can result from impairing the assembly of the eIF4F complexes or preventing the recycling of the ${\text{tRNA}}_{i}^{\text{Met}}$-GTP-eIF2 ternary complex through eIF2α phosphorylation (1). This inhibition of translation leads to polysome disassembly and the formation of SGs through the binding of RNA-binding proteins to nontranslating mRNAs. Translational arrest is problematic for all viruses as they depend on the host cell protein synthesis machinery for the production of viral proteins. Therefore, many viruses antagonize the stress response pathway and SG formation during infection. The mechanism by which this antagonism occurs varies depending on the viruses and the host cells; several patterns of SG accumulation have been described, including complete inhibition of SGs, stable formation of SGs, and oscillation of SGs (8, 9, 50). In some cases viral gene products can act as antagonists by targeting the virus-activated eIF2α kinases PKR and PKR-like endoplasmic reticulum kinase (PERK) or even by directly modulating the phosphorylation of eIF2α (16, 50–54). In this study, we show that although FCV infection results in the phosphorylation of eIF2α, reflecting the activation of stress pathways, SGs do not accumulate in FCV-infected cells (Fig. 1 and 2). We further demonstrate that FCV infection impairs SG assembly in response to translational arrest driven by eIF2α phosphorylation and also by eIF4F complex disruption (Fig. 3 and 4). This suggests that FCV does not target the stress response signaling pathway but only the downstream formation of SGs.

Several RNA viruses have been shown to express viral effectors that can actively disrupt the accumulation of SGs through sequestration or cleavage of SG components (8, 9). The nonstructural proteins of several alphaviruses redirect SG-nucleating protein into viral replication complexes to disrupt SG accumulation, such as FGDF motifs within Semliki Forest virus (SFV) nsP3, which binds G3BP1 (55–58). The infections of several picornaviruses such as poliovirus, coxsackievirus B3, enterovirus 71, and encephalomyocarditis virus result in the transient formation of SGs (44, 59, 60). Following their early assembly, viral proteinases can disperse SGs by cleaving the SG-nucleating protein G3BP1 (44, 59–61). The calicivirus NS6^Pro^ proteins cysteine proteinases that adopt chymotrypsin-like folds similar to those of 3C proteinases from picornaviruses (62, 63). We therefore hypothesized that FCV infection may result in G3BP1 cleavage. Indeed, the analysis of FCV-infected cells, in two cell lines supporting FCV infection, revealed that G3BP1 undergoes cleavage from 4 hpi (Fig. 5).

To directly show that the FCV NS6^Pro^ is responsible for G3BP1 cleavage, we overexpressed FCV NS6^Pro^ and analyzed the impact on G3BP1 integrity (Fig. 6). Our results demonstrate that FCV NS6^Pro^ cleaves G3BP1, while NS6^Pro^ from another calicivirus, MNV, does not and that the G3BP1 cleavage product differs from the one generated by the PV 3C^Pro^. While the PV 3C^Pro^ cleaves between residues Q325/G326, our mutational analysis identified the FCV NS6^Pro^ cleavage site at E405/V406. By overexpressing the wild-type or noncleavable G3BP1, together with FCV NS6^Pro^, and by challenging cells with sodium arsenite, we demonstrated that preventing NS6^Pro^-mediated G3BP1 cleavage rescues the formation of SGs (Fig. 7). Therefore, this confirms that the cleavage of G3BP1 during FCV infection may act as a major contributor to the inhibition of SG assembly. G3BP1 contains several functional domains, such as an N-terminal NTF2-like domain, central PXXP motifs, and two C-terminal RNA binding domains, with an RRM motif followed by an RGG motif. While PV 3C^Pro^ cleaves off both RNA binding domains, a property conserved in other enteroviruses, FCV NS6^Pro^ cleaves G3BP1 between the RRM and the RGG motifs, targeting only the C-terminal domain. Thus, the modular scaffold of RNA-binding elements within G3BP1 can be differentially targeted by viral proteinase to cleave G3BP1.

To explain the absence of SG inhibition following MNV infection and G3BP1 cleavage by MNV NS6^Pro^, we compared the sequences of the human, feline, and murine G3BP1 proteins (Fig. 6C). The FCV NS6^Pro^ cleavage site is conserved between human and cat, which could explain the impairment of SG assembly during FCV infection. However, murine G3BP1 has at position 405 an alanine instead of the aspartate found in feline and human G3BP1 proteins. The previous determination of the MNV NS6^Pro^ structure in complex with a substrate sequence revealed that three amino acids, H157, T134, and Y143, make extensive contacts with the side chain carbonyl of a glutamate residue at the cleavage site. Therefore, the presence of an alanine in the murine G3BP1 could explain the lack of cleavage by the MNV NS6^Pro^ during infection and, consequently, the ability of MNV-infected cells to assemble SGs in response to arsenite treatment, at least in J774 cells (Fig. 8). However, this does not explain why MNV NS6^Pro^ is unable to cleave G3BP1 when it is expressed in human cells (Fig. 6A). We could speculate that differences in amino acids surrounding the NS6^Pro^ catalytic triad between MNV and FCV could potentially impact the substrate recognition. The recognition of G3BP1 by NS6^Pro^ from different caliciviruses will be the subject of further structural investigation studies beyond the scope of this study. G3BP2 is a G3BP1 homolog with a similar domain organization (64). Like G3BP1, G3BP2 is important for SG formation and is sequestered by chikungunya virus nsP3 to disassemble SGs late in infection (39, 47, 65). Recently, it was shown that G3BP2 is not cleaved by PV 3C^Pro^, questioning the importance of G3BP2 in SG assembly (66). Our study shows that G3BP2 can also be cleaved by FCV NS6^Pro^, suggesting that both G3BP homologs are targeted by FCV NS6^Pro^ (Fig. 6). This observation correlates with the fact that the G3BP1 cleavage site is conserved in human and feline G3BP2 (G3BP1, ^402^FRGE/VRLN^409^; G3BP2, ^396^FRGE/VRLN^403^). However, overexpression of noncleaved G3BP1 is sufficient to restore SG formation under conditions where G3BP2 is cleaved. Therefore, further work will be necessary to dissect the contribution of G3BP2 cleavage to the inhibition of SGs.

Increasing evidence has suggested a functional link between the induction of SGs by viruses and the initiation of innate antiviral responses (8, 19, 67). The observations that there is a correlation between SG formation and IFN production and a reverse correlation between SG formation and viral propagation and that pattern recognition receptors are localized in virus-induced SGs together with viral non-self RNAs together strongly suggest that SGs have an antiviral role and possibly function as a platform to initiate innate antiviral responses (16–18, 59, 67–69). Overall, virus-induced SGs might play dual roles: (i) suppressing viral replication through an inhibition of viral protein synthesis, and (ii) serving as a platform to facilitate IFN production. Our observation that FCV inhibits SG assembly and that NS6^Pro^ cleaves G3BP1 may reflect one of the many strategies that caliciviruses have evolved to control the host antiviral response.
